# Genome-wide association mapping of grain yield in a diverse collection of spring wheat (*Triticum aestivum* L.) evaluated in southern Australia

**DOI:** 10.1371/journal.pone.0211730

**Published:** 2019-02-04

**Authors:** Melissa Garcia, Paul Eckermann, Stephan Haefele, Sanjiv Satija, Beata Sznajder, Andy Timmins, Ute Baumann, Petra Wolters, Diane E. Mather, Delphine Fleury

**Affiliations:** 1 Australian Centre for Plant Functional Genomics and School of Agriculture, Food and Wine, Waite Research Institute, The University of Adelaide, Glen Osmond, SA, Australia; 2 Rothamsted Research, Harpenden, United Kingdom; 3 Corteva Agriscience, New Holland, PA, United States of America; Institute of Genetics and Developmental Biology Chinese Academy of Sciences, CHINA

## Abstract

Wheat landraces, wild relatives and other ‘exotic’ accessions are important sources of new favorable alleles. The use of those exotic alleles is facilitated by having access to information on the association of specific genomic regions with desirable traits. Here, we conducted a genome-wide association study (GWAS) using a wheat panel that includes landraces, synthetic hexaploids and other exotic wheat accessions to identify loci that contribute to increases in grain yield in southern Australia. The 568 accessions were grown in the field during the 2014 and 2015 seasons and measured for plant height, maturity, spike length, spike number, grain yield, plant biomass, HI and TGW. We used the 90K SNP array and two GWAS approaches (GAPIT and QTCAT) to identify loci associated with the different traits. We identified 17 loci with GAPIT and 25 with QTCAT. Ten of these loci were associated with known genes that are routinely employed in marker assisted selection such as *Ppd-D1* for maturity and *Rht-D1* for plant height and seven of those were detected with both methods. We identified one locus for yield per se in 2014 on chromosome 6B with QTCAT and three in 2015, on chromosomes 4B and 5A with GAPIT and 6B with QTCAT. The 6B loci corresponded to the same region in both years. The favorable haplotypes for yield at the 5A and 6B loci are widespread in Australian accessions with 112 out of 153 carrying the favorable haplotype at the 5A locus and 136 out of 146 carrying the favorable haplotype at the 6A locus, while the favorable haplotype at 4B is only present in 65 out of 149 Australian accessions. The low number of yield QTL in our study corroborate with other GWAS for yield in wheat, where most of the identified loci have very small effects.

## Introduction

Wheat is the second most important cereal crop worldwide [1]. It is also the most important crop in Australia, with grain production of about 25 million tonnes per year. With a growing world population and increasing consumption per capita, food production needs to increase by 70% to be able to meet the demands projected for 2050[2]. This would require increasing annual wheat yield by at least 1.6% per year, which is substantially higher than historical rates of yield increase for wheat, estimated at about 1% per year. Given that climate change is expected to significantly increase temperatures in wheat production regions [3–4], it is critical to improve the crop’s ability to increase grain yield with less water [5]. This is particularly challenging in regions where drought and heat are endemic, such as southern Australia.

In southern Australia, spring-habit wheat is sown in early autumn (often in May) so that the crop can rely upon autumn and winter rainfall. In spring, rainfall becomes more sporadic and heat waves often occur. This can lead to drought and heat stress at flowering and during grain filling and significant yield losses (reviewed by [6]). This keeps yields in southern Australia relatively low *e*.*g*. a 5-year mean of around 2 t/ha in the state of South Australia [7]. Despite these harsh conditions, a large quantity of wheat grain is produced. For example, in 2017, South Australia produced around 4 million tonnes of wheat.

The genetic improvement of crop plants is achieved by increasing the frequency of favorable alleles (and/or allele combinations) within gene pools. For wheat, the primary gene pool consists of modern cultivars, landraces, synthetic hexaploids and some of wheat’s wild relatives [8]. Although gene transfer inside the primary gene pool is simple, most breeding programs tend to avoid introduction of broader genetic diversity such as landraces and wild relatives since those might bring alleles that are not adapted to modern agronomical practices and reduce yield. Effective use of landraces, wild relatives and other ‘exotic’ sources could be facilitated by information on the association of specific genomic regions with desirable traits, especially if assays for DNA polymorphisms in those regions can be deployed in marker-assisted selection [9–11].

Considerable research has been conducted to detect and map quantitative trait loci (QTL) for yield and yield components using mapping populations derived from crosses between Australian varieties (*e*.*g*. [12–15]) and to detect QTL in water-limited environments (reviewed by Tricker et al. 2018). Here, we report on the results of a genome-wide association study (GWAS) conducted using a wheat panel that includes landraces, synthetic hexaploids and other exotic wheat accessions that might contribute alleles that would increase yield in water-limited environments such as those in southern Australia.

Genome-wide association studies explore historical recombination events by associating genotypes with phenotypes [16]. They rely on linkage disequilibrium (LD): the non-random association between alleles at different loci. The power of GWAS is impacted by the extent of LD between molecular markers and functional loci, the effects of the functional loci and the size and structure of the population that is analyzed. Its resolution depends on the extent to which LD decays with genetic distance and on the density of markers used [16].

Due to the diverse origins and genetic history of accessions that may be used for GWAS, statistical models are used to account for population structure and avoid the detection of false-positive associations [17–18]. Commonly used GWAS methods employ linear mixed models, in which each individual marker is tested for its association with phenotypes [19– 20]. These methods can account for population structure by grouping individuals through principal component analysis or STRUCTURE analysis [17] and/or by accounting for relationships through a kinship matrix that can be estimated from pedigree information or molecular marker data [21–22]. Instead of simply testing whether a particular locus has an effect on the phenotype, this approach tests whether a locus has an effect on the phenotype that is not explained by population structure or genetic background.

In GWAS, the power of QTL detection can be low. To address this limitation, a quantitative trait cluster association test (QTCAT) was proposed [23], which groups markers into clusters of correlated markers, which may or may not be physically linked, and then searches for associations between clusters and phenotypes, rather than between individual markers and phenotypes. This could possibly remove the need to correct for population structure as one part of the phenotypic variation will only be associated with one cluster, due to the simultaneous nature of the testing approach.

Here we describe the detection of genotype: phenotype associations for traits that were measured in a panel of over five hundred bread wheat accessions grown in southern Australia. We applied and compared linear mixed models and QTCAT approaches for the detection of these associations.

## Material and methods

### Germplasm and phenotyping

The germplasm evaluated here consisted of a panel of 568 accessions of spring wheat originating from 36 countries. This panel included advanced cultivars, landraces and synthetic hexaploids. Seeds were obtained from INRA, CIMMYT and the Australian Grains Genebank (AGG) (S1 Table).

Two field trials were grown during the 2014 and 2015 seasons under rainfed conditions at Tarlee, South Australia (34.281295^o^ S, 138.772695^o^ E). Management of pests and diseases and fertilizer applications followed local practices. Rainfall data were collected from a station located less than 5 km from the field site. Temperature data were collected from a station at Roseworthy, South Australia, around 30 km from the field site. Plots were six rows wide by 4 m long, with 22.5 cm spacing between rows within plots.

The 2014 trial included 744 plots arranged in 62 columns by 12 rows. A total of 504 accessions were grown, including 239 with two replicates and 265 with one replicate (with one empty plot) in a partially replicated (p-rep) design [24]. Of the 504 accessions, 502 had been genotyped. In 2015, 553 accessions were grown in a partially replicated design with 503 accessions in two replicates and 50 accessions in one replicate. In this experiment, the 1056 plots were arranged in 88 columns by 12 rows. Of the 553 accessions, 550 had been genotyped.

In both 2014 and 2015, plant height (PH, cm) was measured for three randomly chosen plants per plot, from the soil surface to the tip of the spike excluding awns. In 2014, plant growth stages were recorded using the Zadoks scale [25] at 104 (Maturity 1), 118 (Maturity 2) and 140 (Maturity 3) days after sowing. In 2015, plant growth stages were recorded at 106 (Maturity 1), 126 (Maturity 2) and 146 (Maturity 3) days after sowing. In each year, above-ground plant biomass (PB, g) was estimated by weighing plant materials harvested from a quadrat of 50 cm x 2 rows in the middle of each plot, representing 1/24 of the plot. Plant biomass was converted to tonnes per hectare for analysis. Using the materials harvested from these quadrats, the number of spikes (SN) was counted in both years. In 2014, the length of the spike (SL) was measured from the first rachis to the tip of the last spikelet, excluding awns, on four randomly chosen spikes per plot. Plots were machine harvested and the harvested grain was cleaned. The cleaned grain was weighed to estimate grain yield (YLD, t/ha). Harvest index (HI) was calculated for each plot by dividing the total grain yield (YLD) by the total biomass yield (PB). Thousand grain weight (TGW) was measured for one replicate in 2014 and all replicates in 2015, by counting and weighing 500 grains and multiplying by 2.

### Genotyping

A total of 563 accessions were genotyped using the Illumina iSelect 90K SNP array [26]. The raw intensity (.idat) files produced from the Illumina iScan array scanner were imported into the Illumina genotyping software platform (GenomeStudio v2013, polyploid clustering module v1.0.0). The clustering was performed according to the wheat Illumina iSelect 90K genotyping procedure previously described [26]. Samples were clustered using the DBSCAN [27] and OPTICS [28] clustering algorithms and filtered using the GenomeStudio platform for SNP calling. SNPs with minor allele frequencies below 0.05 were removed from the dataset. In total 30,548 polymorphic markers were retained for the 563 genotyped accessions.

Markers for specific loci known to affect plant phenology, plant height and grain weight were genotyped using Kompetitive Allele Specific Polymerase Chain Reaction (KASP) technology. KASP assays for *Vernalization* genes (*Vrn-A1*, *Vrn-D1*), *Reduced Height* gene (*Rht-B1*, *Rht-D1*) and *Photoperiod* genes (*Ppd-A1*, *Ppd-B1* and *Ppd-D1*) were obtained from the CerealsDB website (http://www.cerealsdb.uk.net/cerealgenomics/CerealsDB/kasp_download.php?URL=, S2 Table). Primers to assay SNPs in the grain weight gene, *TaGW2-6B*, (S3 Table) were designed in house using the software Kraken (LGC Limited, London, UK), the available haplotype information [29] and the whole genome sequencing of 16 wheat varieties available through DAWN (Diversity Among Wheat geNomes, [30]). All KASP markers were genotyped using an automated SNPLine system (LGC Limited, London, UK) following instructions from the manufacturer. Genetic map positions for the iSelect 90K SNP markers were obtained from the 90K consensus map [26].

### Genetic structure analysis

Population structure was estimated using a model-based approach implemented in the software ADMIXTURE version 1.23 [31]. Accessions with >5% missing values were removed, and markers pruned according to the observed sample correlation coefficients to mitigate background linkage disequilibrium: we removed SNP markers that had correlation coefficient value > 0.1 with any other SNP within a 50 cM sliding window, advanced by 10 markers [32] and marker order based on the wheat consensus map [26]. In total 4,571 SNP markers and 514 lines were submitted to the analysis of ancestry in the ADMIXTURE. The number of ancestral populations K was chosen based on the evaluation of cross-validation error in the 5-fold cross-validation procedure of ADMIXTURE. In parallel, principal component analysis (PCA) analysis of 4,751 SNP markers and 514 lines was conducted using smartpca, implemented in the software EIGENSOFT version 5.0.1[33].

### Statistical analysis of phenotypic data

Before GWAS analysis, the phenotypic data from both trials for each trait were checked and spatially analyzed using the ASReml [34] package in R. Accession was fitted as a fixed effect and spatial terms were fitted as random effects [35]. The best linear unbiased estimators (BLUEs) of each accession for each trait in each year were used as input data for GWAS. Heritability (H^2^) was estimated using a secondary model with accession as a random effect using the method of [24], for all traits except TGW in 2014, which was measured only on a single replicate. Adjusted heritabilities after removing the effects of four major loci (*Ppd-B1*, *Ppd-D1*, *Rht-B1* and *Rht-D1*) as fixed effects were also estimated.

### Genome wide association study

Two GWAS methods were used: the compressed mixed linear model (CMLM) [20] implemented in the GAPIT R package [22] and the quantitative trait cluster association test (QTCAT) implemented in R [23].

In the GAPIT analysis, we accounted for population structure (Q) through a principal component (PC) analysis and for relationships among individuals through a kinship (K) matrix [36], both using the marker data. For each trait, the optimal number of PCs/covariates to include in GWAS models was determined through model selection using the Bayesian information criterion (BIC), with a maximum of four PCs tested. The significance threshold for marker-trait associations (MTA) was set to p = 0.05 after applying the false discovery rate (FDR, [37]) correction. We identified all MTA below the threshold as significant. Significant MTA that are collocated on the genetic map [26] defined the sequence intervals for candidate genes. The percentage of variation explained by the MTA (R^2^) was calculated as the difference between the R^2^ of the GAPIT model with and without the strongest associated SNP.

In QTCAT, the first step of the analysis was to generate a hierarchical clustering of all markers based on their correlations. Secondly, these clusters were tested for significant associations for each trait along this hierarchy, using a family-wise error rate threshold of α = 0.05. This step was repeated for all clusters showing significant association until none of the clusters of the next lower level was significantly associated anymore or until the single marker level was reached.

Sequences of SNP markers significantly associated with phenotypic traits were aligned to the International Wheat Genome Sequencing Consortium (IWGSC) Chinese Spring (CS) RefSeq v1.0 [38] using BLASTN with an e-value cutoff of 10^−40^ in order to find their putative locations on the wheat genome. DAWN (Diversity Among Wheat geNomes, [30]) was used to visualize the polymorphisms and gene content in the QTL regions across 16 sequenced wheat varieties [39].

## Results

### Phenotypic traits

In 2014, rainfall at the trial site was higher than average early in the year (February, May and June) but less than average later in the year (from August through December) (S1 Fig). In 2015, there were closer to average rainfall conditions at Tarlee throughout the growing season. The maximum temperatures throughout the season were very similar in both years, reaching 30°C at the end of the crop cycle (S1 Fig).

A summary of the phenotypic traits is given in Table 1 and the raw phenotypic data is available from Figshare (DOI: 10.25909/5becfa45c176f). Heritability estimates were quite high for most traits, including grain yield for which heritability was estimated at 70% and 85% in 2014 and 2015, respectively. This is not surprising, given the wide range of genetic material used. As expected in a dry and hot climate, grain yield was positively correlated with Zadoks scores, above ground biomass and HI and negatively correlated with plant height (S4 Table). Correlations for the same traits between 2014 and 2015 trials ranged from 0.349 for plant biomass to 0.887 for Maturity 2 (Table 1).

**Table 1:** Descriptive statistics of the wheat diversity panel in field trials.

	2014 trial						2015 trial						
Trait	Min	Mean	Max	N	H^2^	H^2^—major genes	Min	Mean	Max	N	H^2^	H^2^—major genes	Correlation*
Plant height	50.00	89.97	135.00	743	75.7	66.1	40.00	92.00	135.00	1055	76.5	69.4	0.774
Zadoks 1	26.00	38.47	55.00	743	72.6	70.9	29.00	39.28	57.00	1054	71.6	68.7	0.661
Zadoks 2	37.00	54.52	70.00	743	95.1	93.0	33.00	57.56	70.00	1054	91.9	88.0	0.887
Zadoks 3	46.00	76.57	86.00	743	70.3	63.6	50.00	80.12	89.00	1054	49.6	42.0	0.683
Biomass	4.62	10.74	19.18	743	12.4	7.8	4.31	10.29	18.09	1011	28.3	23.1	0.349
No of spikes	35.00	86.73	159.00	743	38.1	35.6	25.00	102.10	184.00	1011	49.1	48.8	0.423
Spike length	4.00	9.13	14.00	743	63.5	61.8							
Yield	0.30	2.56	4.30	742	70.1	55.5	0.07	2.01	3.85	1050	85.6	76.1	0.679
HI	0.02	0.25	0.59	742	43.4	29.9	0.01	0.20	0.43	1005	68.5	53.8	0.491
TGW	20.46	33.54	46.30	499			16.40	31.52	54.00	1020	89.2	87.1	0.579

HI: harvest index; TGW: thousand grains weight; Correlation: Pearson r^2^ coefficient between 2014 and 2015 trials; N: number of accessions x replicates.

*All correlations were highly significant: p<0.0000001.

### Genotyping and genetic structure of the spring wheat panel

A total of 30,548 SNP markers were polymorphic across the diversity panel, with 6,510 of these identical to other markers in the dataset, leaving 24,038 unique markers. Map positions were available for 24,982 markers [26], from which 9,597 mapped to the A genome, 12,724 to the B genome and 2,661 to the D genome. All KASP assays tested were polymorphic among the accessions and were included in the GWAS. The genotyping data is available from Figshare (DOI: 10.25909/5becfa45c176f).

**Fig 1:**
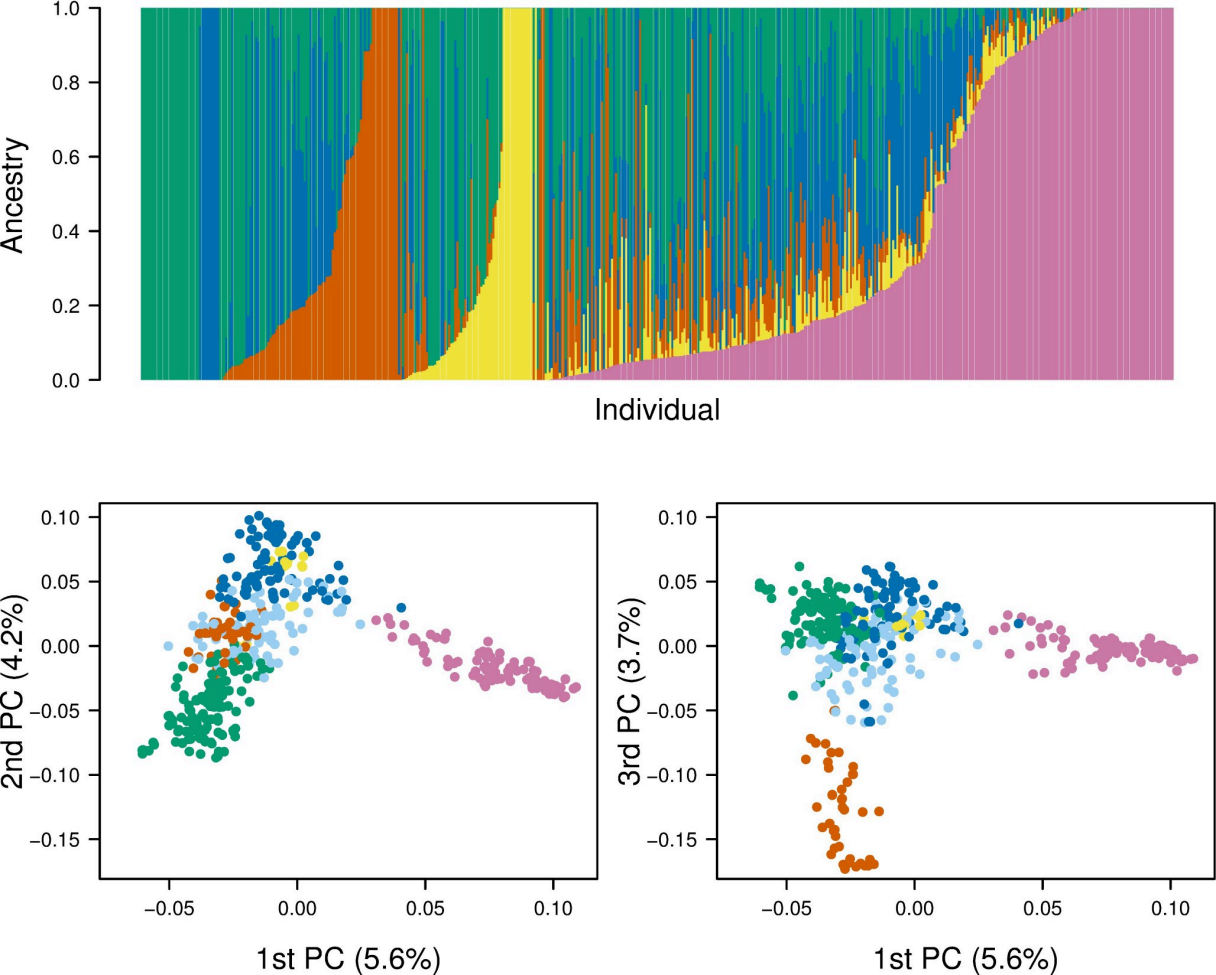
Genetic structure of the diversity panel. A—the ancestral fractions estimated for each accession in software ADMIXTURE for the number of ancestral populations K = 5, indicated with five colors. B—the results of the principal component analysis of the genotyping data, superimposed colors indicate the dominant ancestral fraction of the genotypes (value ≥ 0.5) and are equivalent to the colors used in A, except for light blue which indicates the genotypes for which no ancestral fraction was dominant.

The evaluation of cross-validation errors calculated for varying numbers of ancestral population K in the ADMIXTURE analysis showed that the error levelled off at K = 4 but continued to decrease somewhat for values of K > 4. The ancestral fractions estimated in ADMIXTURE for the number of ancestral populations K = 4 are shown in Fig 1A. Based on the estimated ancestral fractions, the accessions were assigned to five clusters such that samples with the same dominant ancestral fraction (value ≥ 0.5) were assigned to the same cluster and samples for which all four ancestral fractions were < 0.5 were assigned to a fifth cluster. These cluster assignments were superimposed on the results of the PCA analysis (Fig 1B). Together, these results show varying degrees of mixed ancestry for most accessions.

Pairs of accessions that showed very high levels of genetic similarity (> 95% of markers with identical scores) were checked against pedigree information, and other known information about the background of the accessions. For example, two CIMMYT lines with very similar pedigrees differed at only 10 out of 23,833 markers indicating that they are almost certainly the same accession. Due to uncertainty about the true identity of these lines, both members of such pairs were removed from the GWAS analysis. This left subsets of 458 and 502 accessions for the 2014 and 2015 analyses, respectively.

### Marker-trait associations using CMLM and QTCAT

We tested the commonly used CMLM approach implemented in GAPIT and the recently developed QTCAT approach. In GAPIT, we accounted for population structure through PC analysis and model selection to determine the optimal number of PCs to include for each trait. A kinship matrix was also included in the GAPIT model to account for the relationships among individuals. No preference was given to a single method, so Table 2 shows the comprehensive list of significant marker trait associations (MTA) found by either method. QTCAT does not calculate the allelic effect thus Table 2 shows the percentage of variation explained by the MTA (R^2^) for significant associations found by GAPIT. We also show GAPIT’s allelic effect for associations found with QTCAT for which the GAPIT p-value was below 0.15. When an MTA was found with both methods, the p-value is given for both. A list of all significant markers is given in S5 Table.

**Table 2:** Marker-trait associations (MTA) detected by GWAS using QTCAT and GAPIT.

						p-value		
Trait	MTA	Chromosome	Number of markers associated (strongest MTA)	Corresponding gene	Position (cM)	QTCAT	GAPIT	R^2^ (%)*
**2014 trial**								
Zadoks 1	*QMat1*.*aww-2D*	2D	1 (wMAS000024)	*Ppd-D1*	na		0.024	5.43
Zadoks 2	*QMat2*.*aww-2D*	2D	1 (wMAS000024)	*Ppd-D1*	na	<0.001	<0.001	5.72
Zadoks 3	*QMat3*.*aww-2A*.*1*	2A	1 (Tdurum_contig10785_2433)		26.97		0.047	2.91
	*QMat3*.*aww-2A*.*2*	2A	1 (Kukri_rep_c90581_382)		106.30	0.043		
	*QMat3*.*aww-4B*	4B	2 (CAP12_c1416_177)		75.65–76.34		0.047	2.90
	*QMat3*.*aww-5B*	5B	1 (Excalibur_c72450_483)		182.15		0.046	3.02
	*QMat3*.*aww-6A*	6A	1 (BobWhite_c5782_825)		65.98	0.03		
Plant height	*QPh*.*aww-4D*	4D	1 (wMAS000002)	*Rht-D1*	na	0.05	<0.001	8.94
	*QPh*.*aww-6A*	6A	2 (BS00023627_51)		62.53–62.95	0.02		
Spike length	*QSl*.*aww-4D*	4D	4 (BS00064002_51)		94.22	<0.001	0.072	4.31
Number of spikes	*QSn*.*aww-4B*	4B	5 (RAC875_c39226_372)		71.29	<0.001		
Harvest index	*QHi*.*aww-2D*	2D	1 (wMAS000024)	*Ppd-D1*	na	0.012		
Yield	*QYld*.*aww-2D*	2D	1 (wMAS000024)	*Ppd-D1*	na	0.001	0.043	3.63
	*QYld*.*aww-6B*.*1*	6B	3 (GENE-4566_348)		76.07	0.002		
**2015 trial**								
Zadoks 1	*QMat1*.*aww-2D*	2D	1 (wMAS000024)	*Ppd-D1*	na	<0.001	<0.001	6.43
Zadoks 2	*QMat2*.*aww-2A*^◆^	2A	1 (wsnp_BF145580A_Ta_2_2)		na	0.007		
	*QMat2*.*aww-2D*	2D	1 (wMAS000024)	*Ppd-D1*	na	<0.001	<0.001	3.78
Zadoks 3	*QMat3*.*aww-2D*	2D	1 (wMAS000024)	*Ppd-D1*	na	0.001	0.110	2.65
	*QMat3*.*aww-3B*	3B	1 (TA005793-0515)		67.78	0.018		
	*QMat3*.*aww-7B*	7B	1 (BS00111144_51)		52.18		0.044	3.19
Plant height	*QPh*.*aww-4A*	4A	1 (BS00059503_51)		108.72		0.042	3.64
	*QPh*.*aww-4D*	4D	1 (wMAS000002)	*Rht-D1*	na	<0.001	<0.001	8.94
Plant biomass	*QPb*.*aww-4A*	4A	1 (Kukri_c74409_199)		40.27	0.044		
	*QPb*.*aww-5D*	5D	2 (BS00023151_51)		207.33	0.044		
Thousand grain weight	*QTgw*.*aww-4B*	4B	3 (RFL_Contig5365_79)		62.56–62.92	0.029		
Harvest index	*QHi*.*aww-3B*	3B	2 (wsnp_JD_c6974_8084752)		74.22	0.036		
	*QHi*.*aww-6B*	6B	2 (BobWhite_c27364_124)		116.55	0.025		
	*QHi*.*aww-Un*	Un	1 (wsnp_Ex_c6129_10723211)		na	0.029		
Yield	*QYld*.*aww-2D*	2D	1(wMAS000024)	*Ppd-D1*	na	0.004	0.044	1.86
	*QYld*.*aww-4B*	4B	2 (IAAV971)		55.96–57.49		0.017	2.20
	*QYld*.*aww-5A*	5A	4 (Excalibur_c27558_298)		137.98		0.017	2.30
	*QYld*.*aww-6B*.*2*	6B	3 (Kukri_c3292_670)		76.20	0.029		
	*QYld*.*aww-Un*	Un	1 (wsnp_Ex_c6129_10723211)		na	<0.001		

*R^2^ from GAPIT

^**◆**^ Position based on BLAST results only

Markers that showed significant associations with traits were aligned to the Chinese Spring reference sequence (RefSeq v1.0) and BLASTN results are shown in S6 Table. In most cases, positions in the physical map were in agreement with genetic map positions. Some of the markers that had not been genetically mapped could be assigned positions in the physical map.

Significant MTA were found for all traits in at least one trial with a total of 14 MTA in the 2014 trial and 19 in the 2015 trial. The *Ppd-D1* locus on chromosome 2D was associated with Zadoks score and yield in both trials, and with harvest index (*Qhi*.*aww-2D*) in 2014, while the *Rht-D1* locus on chromosome 4D was associated with plant height in both trials (Table 2).

Plant biomass in the 2015 trial was associated with two collocated markers on chromosome 5D and a marker on chromosome 4A (Table 2) that formed part of the same cluster in QTCAT. BLAST results for the 5D markers show that the most significant hit was on chromosome 4A around 570 Mbp distant from *QPb*.*aww-4A* (S5 Table). Hence it is not clear from the information available where the true location of this QTL is. Either of the two regions on 4A or the region on 5D remain possibilities.

Spike length and spike number in the 2014 trial were associated with regions on chromosomes 4D (*QSl*.*aww-4D*) and 4B (*QSn*.*aww-4B*), respectively (Table 2). The positions on the physical map for *QSl*.*aww-4D* (at 455 Mbp on chromosome 4D) and *QSn*.*aww-4B* (at 447 Mbp on chromosome 4B) suggest that these regions might be homeologous (S6 Table).

Thousand grain weight and harvest index in the 2015 trial were associated with regions on chromosomes 4B (*QTgw.aww-4B*), 3B (*QHi.aww-3B*) and 6B (*QHi*.*aww-6B*) (Table 2). Although thousand grain weight and harvest index are yield components, neither of these regions were associated with grain yield.

The marker wsnp_Ex_c6129_10723211, which was not included in the consensus map of Wang et al. (2014), was significantly associated with harvest index and yield in 2015. The BLAST results for this marker show two possible positions on the physical map on chromosomes 3B and 4B, with very similar e-values.

### QTL for yield *per se*

Overall, we found five yield QTL, of which one was linked to *Ppd-D1* on chromosome 2D (Table 2). The other four, which can be considered as QTL for yield *per se* (i.e. not related to phenology) are on chromosomes 4B (*QYld*.*aww-4B*), 5A (*QYld*.*aww-5A*) and 6B (*QYld*.*aww-6B*.*1* and *QYld*.*aww-6B*.*2*). We used DAWN [30] to visualize the yield QTL regions across 16 sequenced wheat accessions [39]. DAWN [30] aligns reads from the whole genome sequencing of 16 wheat accessions [39] onto the Chinese Spring reference genome sequence, allowing to investigate genotypic differences between wheat accessions at the level of whole chromosomes down to individual genes. All but two of these accessions (Volcani and Xiaoyan-54) were included in our GWAS panel.

**Fig 2:**
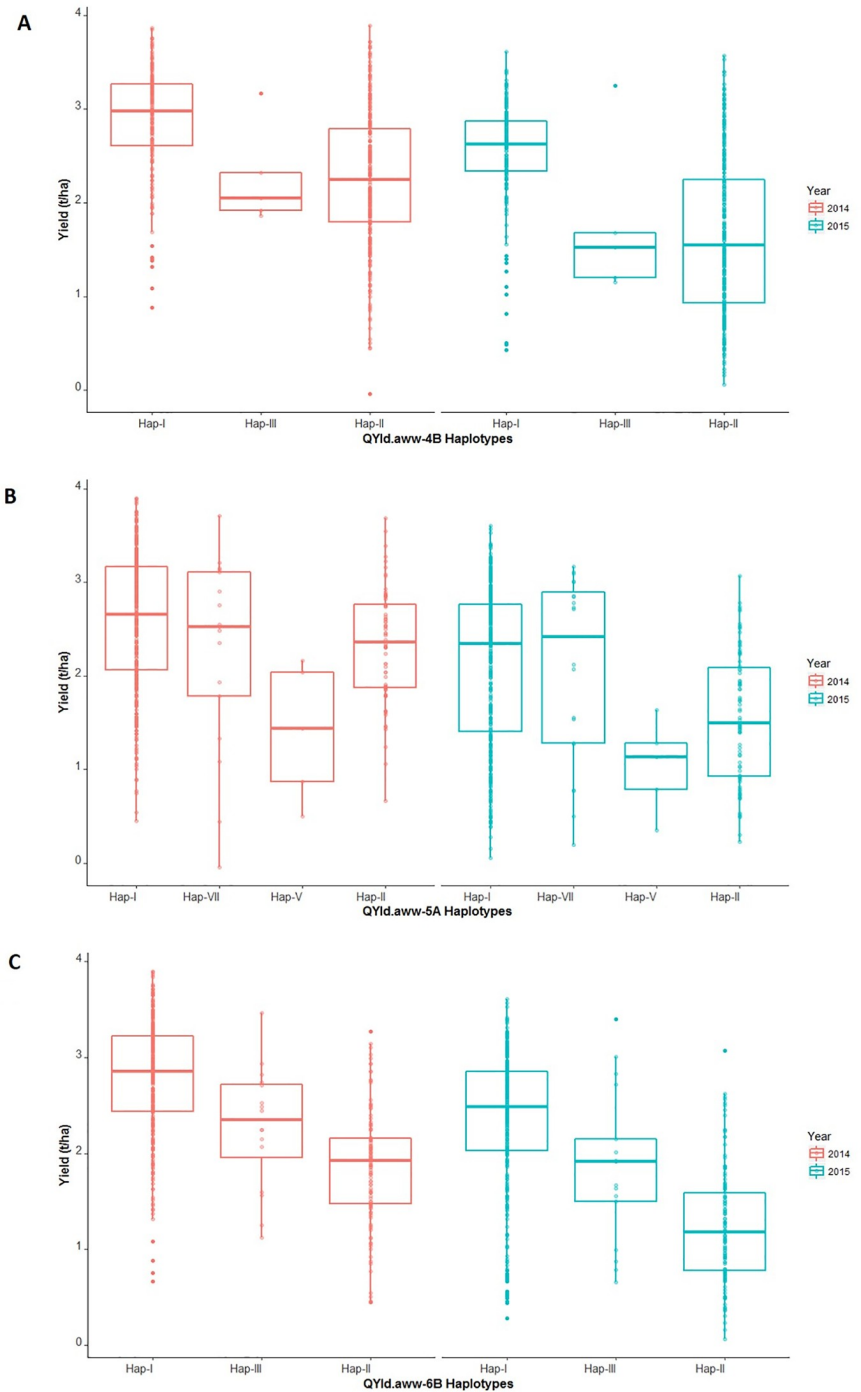
Boxplot of yield in 2014 and 2015 for accessions with different alleles at yield QTL. A–*QYld*.*aww-4B*, B–*QYld*.*aww-5A* and C–*QYld*.*aww-6B*.

The *QYld*.*aww-4B* peak is located in a 2 cM interval on the genetic map [26] that is delimited by the markers wsnp_Ku_c28756_38667953 and IAAV971. This interval corresponds to a 2,727 Kbp region in the RefSeq v1.0. In our panel, some accessions have favorable alleles at both markers (haplotype I), some have unfavorable alleles at both markers (haplotype II) and some have favorable alleles at wsnp_Ku_c28756_38667953 but not at IAAV971 (haplotype III). We compared the phenotypic data among these three haplotypes (Fig 2A). Accessions carrying the favorable allele at both markers yielded significantly more than accessions with either of the other two haplotypes. Many accessions, including Chinese Spring and many Australian varieties, did not carry the favorable alleles at either of these markers (S7 Table).

**Fig 3:**
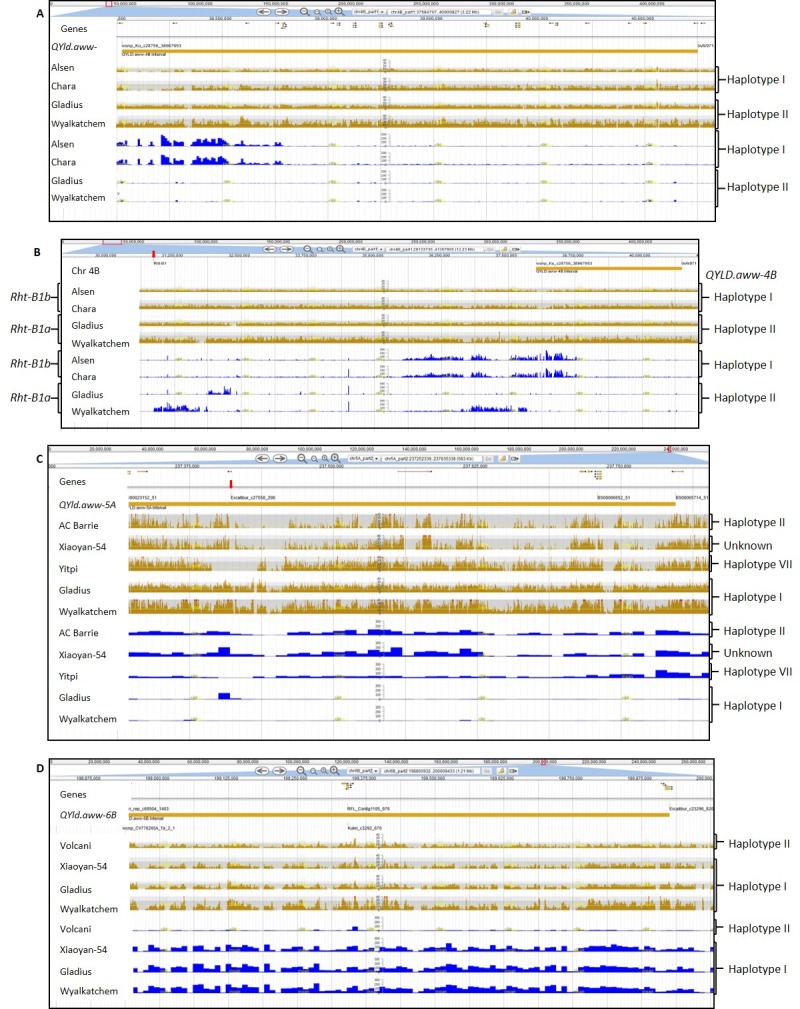
DAWN screenshot of the three yield QTL regions aligned to Chinese Spring reference sequence, RefSeq v1.0. The “Genes” track shows the high confidence genes according to the RefSeq annotation v 1.0. The yellow tracks show the number of reads from a wheat accession that aligns to RefSeq v1.0 at each position of the genome. The blue tracks show the variant density (SNP per 10 Kb–log scaled) between reads from a wheat accession and RefSeq v1.0. **(A)** The “*QYld*.*aww-4B*” track shows the QTL interval and the position of the significantly associated markers. **(B)**–The “Chr4B” track shows the region on chromosome 4B containing *QYld*.*aww-4B* and *Rht-B1*. Different patterns across the blue tracks in the *QYld*.*aww-4B* and *Rht-B1* regions indicate that these two regions segregate independently. **(C)**–The “*QYld*.*aww-5A*” track shows the QTL interval and significantly associated markers. The red arrow indicates the region of the marker Excalibur_c27558_298. **(D)**–The “*QYld*.*aww-6B*” track shows the QTL interval and the significantly associated markers.

Fig 3 shows the level of similarity in genomic sequence between different wheat accessions and the RefSeq v1.0 genome. Accessions with similar profiles such as Alsen and Chara carry the same haplotype at *QYld*.*aww-4B*, in contrast with Gladius and Wyalkatchem which carry the haplotype II (Fig 3A). The sequence polymorphisms observed in DAWN at the two *QYld*.*aww-4B* markers confirmed that out of the 14 sequenced varieties included in our panel, Alsen, Pastor, Baxter, Chara and H45 carry the favorable allele at the marker IAAV971, which seems to be the marker with the larger phenotypic effect (Fig 2A). The dwarfing gene *Rht-B1* is also located on chromosome 4B but ~7 Mbp apart from the *QYld*.*aww-4B* (Fig 3B) and *Rht-B1* was not significantly associated with yield. We also looked at the number of high confidence genes in the QTL interval using the RefSeq v1.0. There were 23 predicted genes with high confidence in the ~2.7 Mbp *QYld*.*aww-4B* region (S8 Table).

*QYld*.*aww-5A* covered four markers collocated on the genetic map [26] and in a 479 Kbp interval on the RefSeq v.1.0 (Fig 3C). We found seven *QYld*.*aww-5A* haplotypes in our panel (S9 Table). Most accessions, including Chinese Spring, carry the favorable alleles at all four markers (S9 Table). Other haplotypes were represented by only one accession, making it hard to estimate their effects (those were excluded from Fig 2). There were five accessions carrying the favorable allele only at Excalibur_c27558_298 and they yielded as low as those carrying only unfavorable alleles (Fig 2B). In contrast, accessions carrying the favorable allele only at BS00023152_51 yielded as much as those containing the favorable allele at all markers (Fig 2B). Polymorphisms observed in DAWN for three of the *QYld*.*aww-5A* markers (BS00023152_51, BS00066652_51 and BS00065714_51) confirmed that Yitpi carries the favorable allele only at BS00023152_51, Xioyan-54 carries the favorable allele only at BS00066653_51 and that AC Barrie carries only unfavorable alleles (S9 Table). All other accessions available in DAWN carried the favorable alleles at all loci. In the region containing the marker Excalibur_c27558_298 the only reads that aligned well to RefSeq v1.0 were those from accessions that carry the favorable allele at all three *QYld*.*aww-5A* markers. This indicates that accessions carrying alternative alleles are so different from Chinese Spring that reads cannot align to each other (Fig 3C). Within the ~480 Kbp QTL region on chromosome 5A, eight high-confidence genes have been predicted (S10 Table).

The estimated positions of *QYld*.*aww-6B*.*1*, which was detected based on yield data from 2014, and *QYld*.*aww-6B*.*2*, based on 2015 yield data, are just 6.13 cM apart [26]. As these two QTL also physically overlapped, with five of six significant markers anchoring within a region of 995 Kbp on the RefSeq v1.0 (Fig 3D), we considered them as a single locus (*QYld*.*aww-6B*). Using the six markers, we detected four haplotypes (S11 Table). One of the haplotypes was represented only by the accession Precoce du Japon. Chinese Spring and most of the landraces carried the unfavorable allele at all six markers in the interval as shown for the variety Volcani on DAWN profile (Fig 3D). Most varieties (including Xiaoyan-54, Gladius, Wyalkatchem, Fig 3D) carried the favorable allele at all markers giving them a yield increase of around 1 t/ha compared to accessions carrying unfavorable alleles (Fig 2C, S11 Table). A total of 17 accessions carried the favorable alleles at three out of six markers and showed intermediate yield performance in both trials (Fig 2C, S11 Table). We found nine predicted genes in the 1 Mbp region of *QYld*.*aww-6B* (Fig 3D, S12 Table).

**Fig 4:**
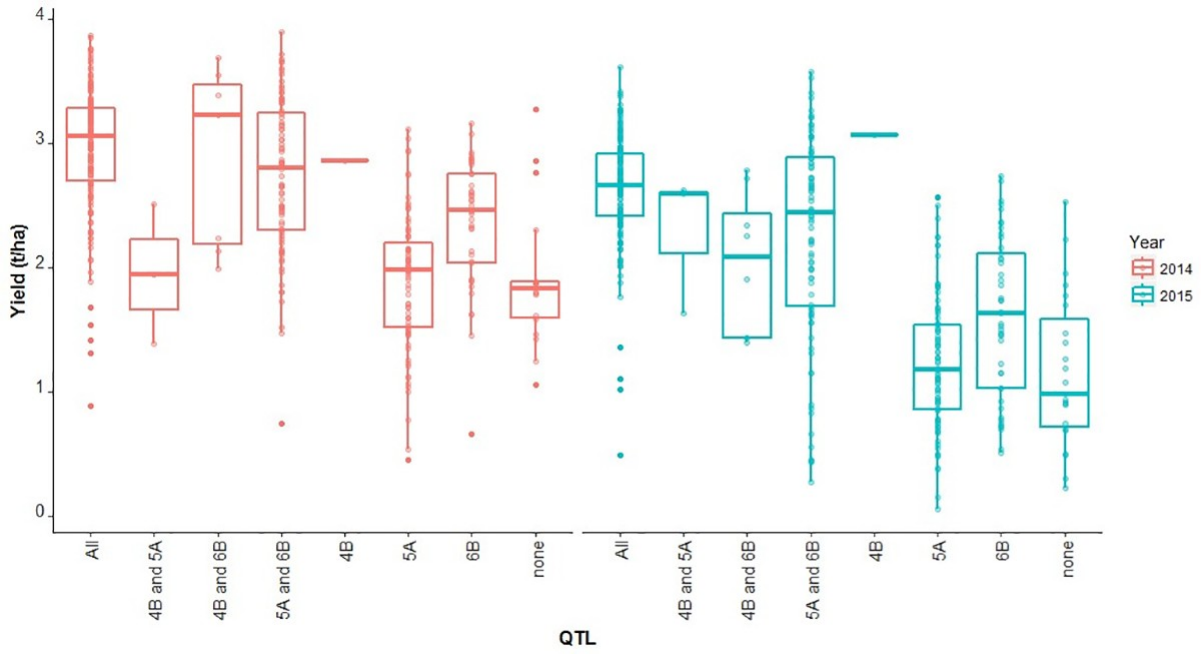
Boxplot of yield in 2014 and 2015 for accessions carrying favorable haplotypes at different yield QTL combinations.

Classification of the accessions according to their haplotypes at the yield QTL on 4B, 5A and 6B provided 19 classes, with between two and 288 accessions per class. We focused on the haplotypes made of only favorable alleles or only unfavorable alleles, which comprised eight classes: three classes of accessions carrying the favorable alleles at single QTL, one class of accessions containing the favorable alleles at all three QTL, one class of accessions containing unfavorable alleles at all three QTL and the other three classes of accessions containing favorable alleles at different combinations of two QTL. Comparison of the yields of these eight genotypic classes (Fig 4) shows that accessions containing two favorable alleles (at *QYld*.*aww-4B* and *QYld*.*aww-6B* in 2014, and at *QYld*.*aww-4B* and *QYld*.*aww-5A* in 2015) yielded as much as those containing the favorable allele at all three yield QTL. The comparison of the effect of the individual QTL is complicated by the fact that only two accessions carry only *QYld*.*aww-4A*. *QYld*.*aww-6B* in isolation had an effect larger than that of *QYld*.*aww-5A* (Fig 4).

## Discussion

Many studies aimed at mapping yield related QTL in wheat biparental populations in dry and hot climates (reviewed in [6]), and there have been several attempts to map yield loci through GWAS [40–43]. Domestication and selection create a genetic bottleneck by removing low-frequency alleles, decreasing variation, increasing LD and creating linkage drag [44]. This is particularly true for self-pollinating species like wheat. By exploiting broad genetic diversity, GWAS attempts to overcome this problem.

Most GWAS methods use correction for genetic structure to reduce false marker-trait associations. The standard correction for genetic structure is a very stringent restriction on a GWAS analysis that often leads to a reduction of power in detecting true association. If a trait is highly correlated with genetic structure, then the significance of true positive decreases [44]. This is particularly problematic for populations with a strong genetic structure such as wheat and barley. The QTCAT method has been developed to overcome this issue [23]. Instead of correcting for genetic structure, it considers the correlation between markers to detect multiple marker-trait association simultaneously. To date, this method has been used in Arabidopsis, where the authors found 50 significant quantitative trait clusters for flowering time using QTCAT compared to none when using a standard threshold in a linear mixed model analysis [45].

Here, we studied a panel of around 550 diverse wheat accessions in two field trials and identified a total of 25 MTA using QTCAT and 17 MTA using the GAPIT approach. These results are more consistent with the comparison between methods in a simulation study [23]. This study identified an average of 8.62 loci (8.48 true loci) using QTCAT compared to 12.45 loci (8.52 true loci) using a linear mixed model with a false discovery rate correction.

**Fig 5:**
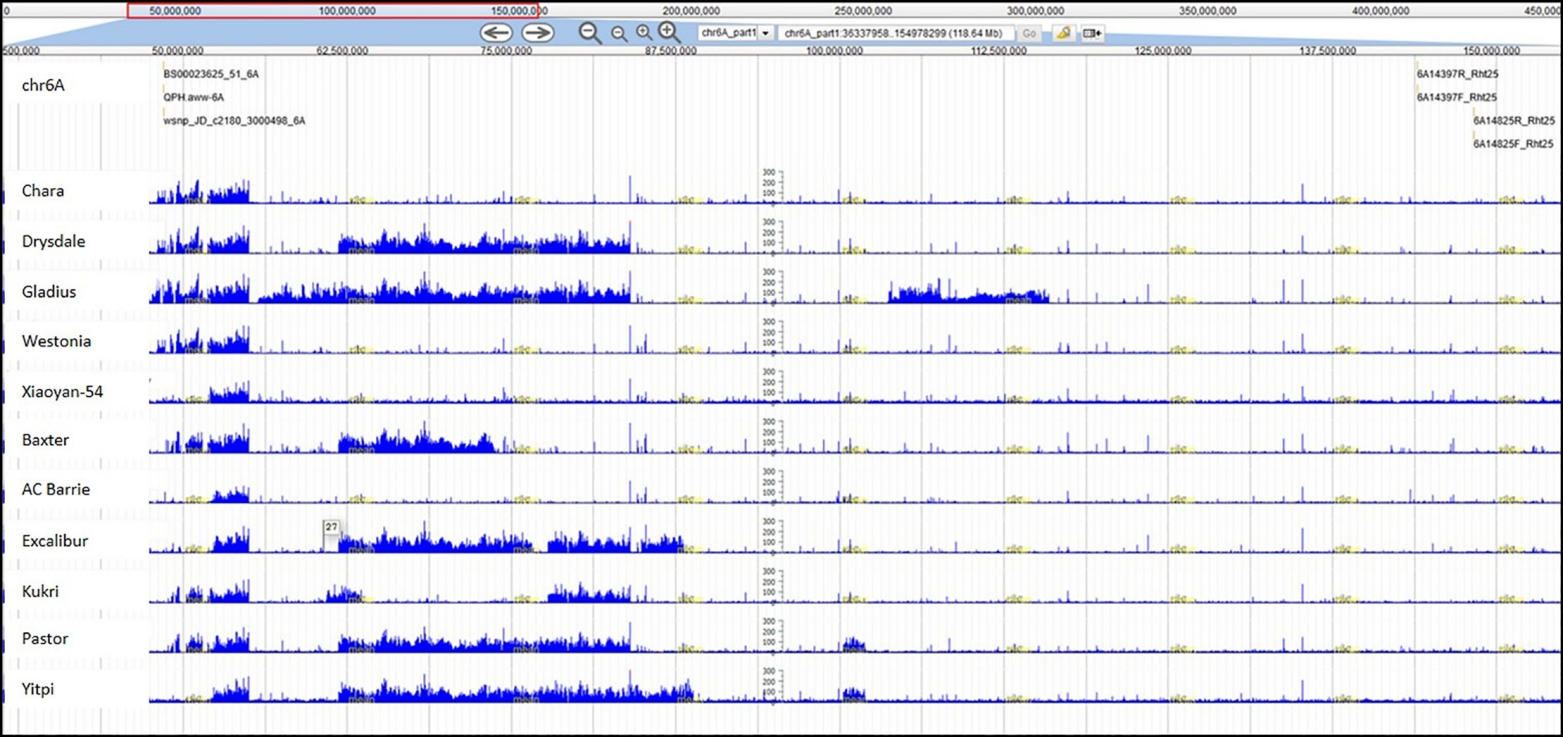
DAWN screenshot of chromosome 6A region containing *QPh*.*aww-6A* and *Rht-25* loci on Chinese Spring RefSeq v1.0. The “Genes” track shows the high confidence genes according to the RefSeq annotation v 1.0. The blue tracks show the variant density (SNP per 10 Kb–log scaled) in comparison to RefSeq v1.0 for 11 wheat accessions; the different profiles across this region indicates that *QPh*.*aww-6A* and *Rht-25* segregate independently.

Some of the marker-trait associations detected here involve polymorphisms within well-known genes that are already used routinely in marker assisted selection. QTL for Zadoks growth stage, yield and harvest index corresponded to the phenology gene *Ppd-D1* on chromosome 2D. This gene has been shown to affect grain yield, especially in hot and dry environments where plants can escape the stress by flowering earlier in the season [46– 47]. Unsurprisingly, plant height was associated with the dwarfing gene *Rht-D1* in both trials. Although *Rht-B1* was also segregating in our panel, it was not significantly associated with plant height after correcting for structure; as the panel is structured in clusters that differ at *Rht-B1*, correction for structure removed *Rht-B1* effects. We also found a locus associated with plant height on chromosome arm 6AS. *QPh*.*aww-6A* is located around 95 Mbp from the recently identified *Rht25* locus, which affects plant height, heading time and spike development [48]. We looked at these two 6AS regions in DAWN [30] and, although it is hard to tell if they are linked to the same causal gene, we can see that the accessions differ in haplotypes across the *QPh*.*aww-6A* and the *Rht-25* regions (Fig 5), indicating that these loci segregate independently.

The favorable haplotype for *QYld*.*aww-4B* was present in fewer than half of the accessions included in our panel. Most of the accessions carrying the favorable haplotype at this QTL came from CIMMYT with 95% of them also carrying the dwarfing allele at *Rht-B1*. 86% of the accessions that carry the unfavorable *QYld*.*aww-4B* haplotype carry the wild-type allele at *Rht-B1*. The co-segregation between *QYld*.*aww-4B* and *Rht-B1* indicates that the favorable haplotype for this QTL could have been introduced from CIMMYT material (S6 Table) together with *Rht-B1*. Since the *Rht-B1* effect is confounded with the effect of population structure in this panel, it is possible that *Rht-B1* is the causal gene for this QTL. CIMMYT material was extensively used around the world and in Australia, especially after the Green revolution in the 70’s, to introduce the semi-dwarf phenotype to local breeding programmes [49].

We also found a QTL for TGW on chromosome 4B (*QTgw*.*aww-4B*) but we could not locate this QTL on the physical map because the BLAST results only returned hits on chromosomes 4A, 4D and unassigned contigs (S6 Table). *QTgw*.*aww-4B* segregates independently from both *QYld*.*aww-4B* and *Rht-B1*, with around half of the accessions that carry the favorable allele at *QTgw*.*aww-4B*, carrying the dwarfing allele at *Rht-B1* (50.5%) or the favorable alleles at *QYld*.*aww-4B* (47%). A previous study using 192 wheat accessions from southwest China found four loci associated with TGW on chromosome 4B [50]. One of these loci (BS00022090_51) was located less than 1 cM from our *QTgw*.*aww-4B* and the other three loci (BS00058659_51, Ex_c57212_719 and wsnp_Ex_c42895_49355806) were located less than 1 cM from our *QSn*.*aww-4B*. A QTL affecting the number of spikes could also affect TGW because of the negative correlation between grain number and grain size. So *QSn*.*aww-4B* may be the same as one of the TGW loci reported [50].

The favorable haplotypes for *QYld*.*aww-6B* and *QYld*.*aww-5A* were very common in our panel, and most of the Australian accessions already carry the favorable alleles at these loci. GWAS in a panel of 123 Pakistani wheat accessions during 2011–2014 seasons under rainfed conditions [42] found a QTL for spike number on chromosome 5A, around 2 cM apart from our *QYld*.*aww-5A* based on the 90K iSelect SNP consensus map [26]. They also found a QTL for yield, harvest index and biomass around 4 cM from our *QYld*.*aww-6B*. Although these QTL [42] do not share any markers with our 5A and 6B QTL (despite using the same genotyping platform), their close proximity in the genetic map could indicate that they are linked to the same causal genes.

Although GWAS has been successfully used in some crops like maize, this methodology may be difficult to apply to complex traits controlled by many small effect QTL like yield in wheat regardless of the methodology used for MTA detection (GAPIT vs QTCAT). In maize, a previous study could identify QTL of grain yield explaining 79% of the genetic variance with a dataset of 244 hybrids, 515k polymorphic SNP and 29 field trials across Europe [51]. A GWAS of 3,816 elite wheat lines from Europe could not find any QTL with an individual contribution > 2.5% despite the high power of detection [52] and the authors concluded that the genetic architecture of grain yield for elite European wheat was too complex to identify stable QTL even in a large population due to many QTL with small effects. Similar results were found using 376 wheat lines [41]. This phenomenon is likely similar for the lines used in this GWAS study and would explain the very low number of yield QTL.

An “omnigenic” model was proposed to explain the missing heritability in GWAS for complex traits [53]. In human genetics, Mendelian diseases are caused by protein-coding changes, while complex traits are mainly caused by noncoding variants affecting gene regulation [53]. The strongest genetic associations found in GWAS explain only a small fraction of the genetic variance. The missing heritability would be due to many causal loci of small effect sizes that do not pass the threshold for significance. The fraction of variance detected by GWAS would be due to a small number of genes, referred as core genes. When core genes are affected by loss of function or other mutations, these genes have a strong effect on the trait. A similar model could be applied to wheat yield with *Ppd*, *Vrn* and *Rht* as core genes. Because regulatory networks are highly interconnected, any expressed gene is likely to affect the regulation of core genes with a small effect size.

The omnigenic model raises questions about current methods for handling the multiple testing issue (such as FDR) which have arisen from the assumption that the majority of loci have an effect size of zero, with only a small number of non-zero effects. Hence new statistical approaches that move away from the paradigm of hypothesis testing may be needed if the majority of genes contribute small non-zero effects [54].

Multiparent populations such as MAGIC (Multi-parent Advanced Generation Intercrosses) and NAM (Nested Association Mapping) populations might be a good alternative to GWAS in the case of grain yield especially in a crop like wheat in which genetic architecture of grain yield may be too complex explained by highly connected regulatory networks. These populations allow to explore more genetic diversity than the traditional biparental populations and have a higher power to detect QTL than GWAS [55–56]. Future studies on yield using MAGIC or NAM populations will be necessary to demonstrate the power of these approaches for complex traits.

## Supporting information

S1 Fig Environmental data from field trials. A) Rainfall in 2014 and 2015 as compared with the historical data (grey boxplot). B) Maximum average temperature in 2014 and 2015 as compared with historical data (grey boxplot).
(PDF)

S2 Fig KASP results obtained with marker wMAS000024 targeting the photoperiod response gene *Ppd-D1*. The three panels represent different accessions. The blue dots represent accessions carrying the *Ppd-D1* insensitive allele, the red dots represent accessions carrying the *Ppd-D1* sensitive allele, the black dots represent negative controls and the pink dots represent accessions for which we were not able to make a confident call.
(JPG)

S3 Fig Manhattan plot of yield in 2014 obtained with GAPIT. The red line represents the FDR corrected p-value of 0.04318, which is the closest to the threshold of FDR corrected p-value = 0.05 obtained in 2014.
(JPEG)

S4 Fig Manhattan plot of yield in 2015 obtained with GAPIT. The red line represents the FDR corrected p-value of 0.04433, which is the closest to the threshold of FDR corrected p-value = 0.05 obtained in 2015.
(JPEG)

S5 Fig Q-Q Plot of yield in 2014 obtained with GAPIT. (PDF)

S6 Fig Q-Q Plot of yield in 2015 obtained with GAPIT. (PDF)

S1 Table List of accessions. (XLSX)

S2 Table KASP marker assisted selection markers. (XLSX)

S3 Table KASP markers developed to target SNP on TaGW2-6B. (XLSX)

S4 Table Correlation among phenotyping traits in field trials of the wheat diversity panel. (XLSX)

S5 Table Marker-trait associations. * Position based on BLAST results.
(XLSX)

S6 Table BLAST results of significant markers against the CS IWGSC RefSeq v1.0. (XLSX)

S7 Table *QYLD*.*aww-4B* haplotypes. Alleles increasing yield are highlighted in blue and alleles decreasing yield are highlighted in yellow. Markers are ordered from left to right based on their position on the CS IWGSC RefSeq v 1.0.
(XLSX)

S8 Table High Confidence genes in the *QYLD*.*aww-4B* interval based on the IWGSC RefSeq Annotation v1.0. (XLSX)

S9 Table *QYLD*.*aww-5A* haplotypes. Alleles increasing yield are highlighted in blue and alleles decreasing yield are highlighted in yellow. Markers are ordered from left to right based on their position on the CS IWGSC RefSeq v 1.0.
(XLSX)

S10 Table High confidence genes in the *QYLD*.*aww-5A* interval based on the IWGSC RefSeq Annotation v1.0. (XLSX)

S11 Table *QYLD*.*aww-6B* haplotypes. Alleles increasing yield are highlighted in blue and alleles decreasing yield are highlighted in yellow. Markers are ordered from left to right based on their position on the CS IWGSC RefSeq v 1.0.
(XLSX)

S12 Table High confidence genes in the *QYLD*.*aww-6B* interval based on the IWGSC RefSeq Annotation v1.0. (XLSX)
