# Supplementary figures and images for: Genome-wide association mapping of grain yield in a diverse collection of spring wheat (Triticum aestivum L.) evaluated in southern Australia
Source: PLoS One. 2019 Feb 4;14(2):e0211730. doi: 10.1371/journal.pone.0211730 (PMC6361508; doi:10.1371/journal.pone.0211730)

A

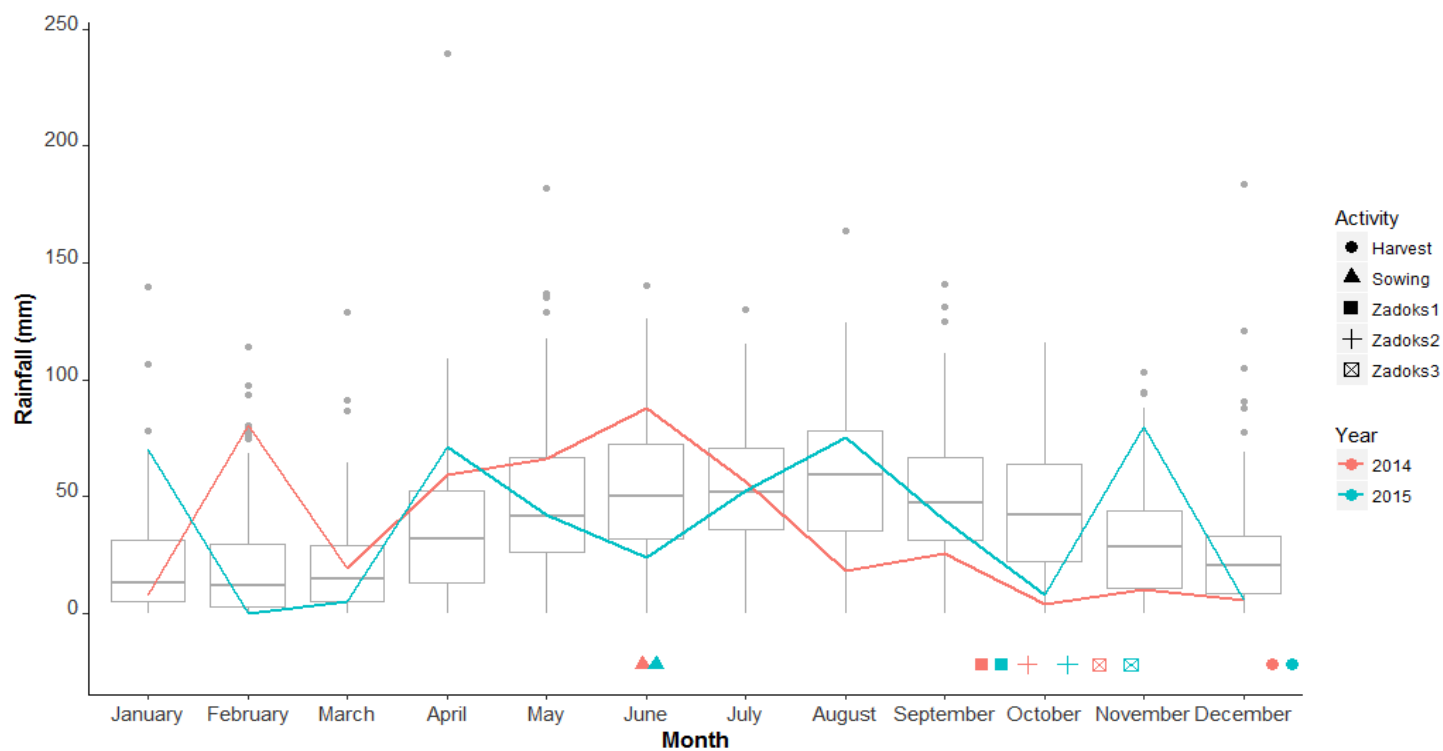

B

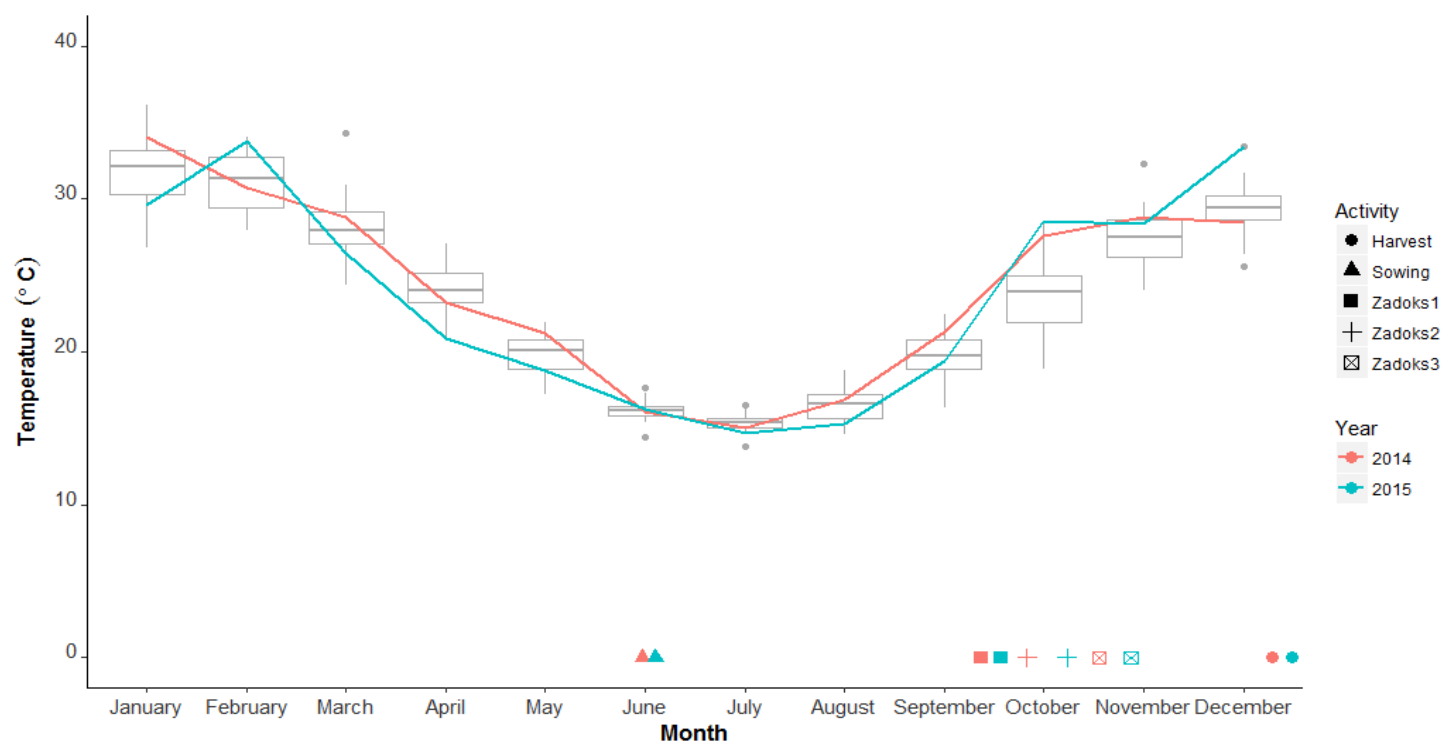

Supplement: S1 Fig — A) Rainfall in 2014 and 2015 as compared with the historical data (grey boxplot). B) Maximum average temperature in 2014 and 2015 as compared with historical data (grey boxplot). (PDF) [file pone.0211730.s001.pdf]

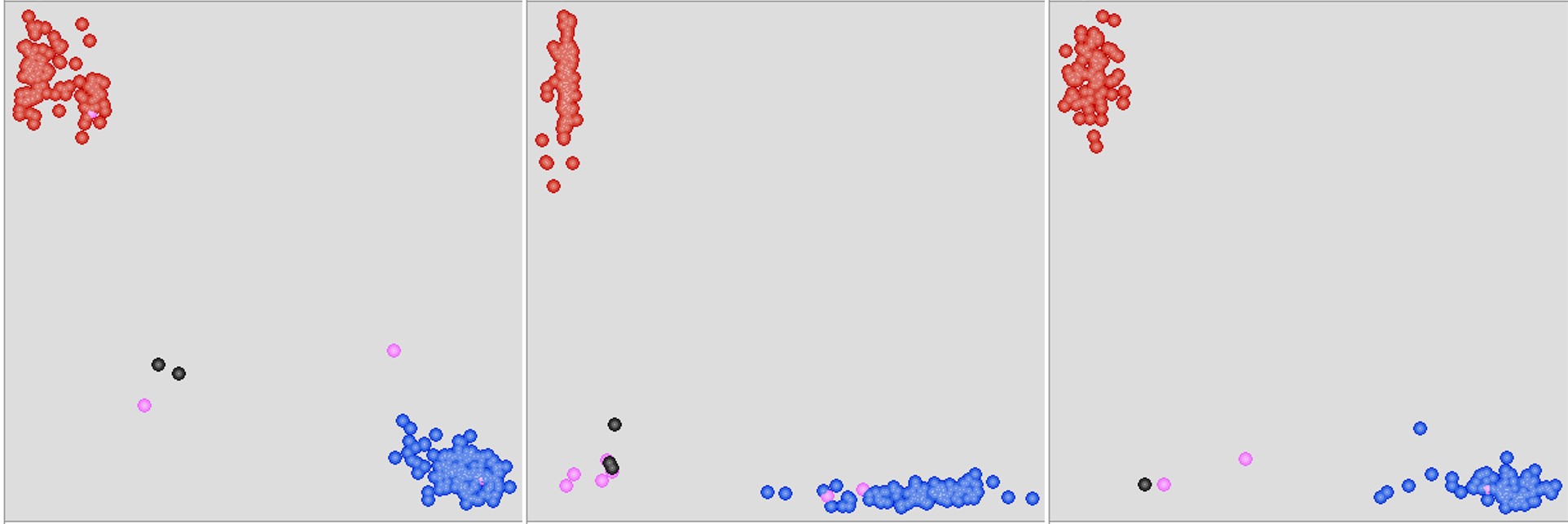

Supplement: S2 Fig — The three panels represent different accessions. The blue dots represent accessions carrying the Ppd-D1 insensitive allele, the red dots represent accessions carrying the Ppd-D1 sensitive allele, the black dots represent negative controls and the pink dots represent accessions for which we were not able to make a confident call. (JPG) [file pone.0211730.s002.jpg]

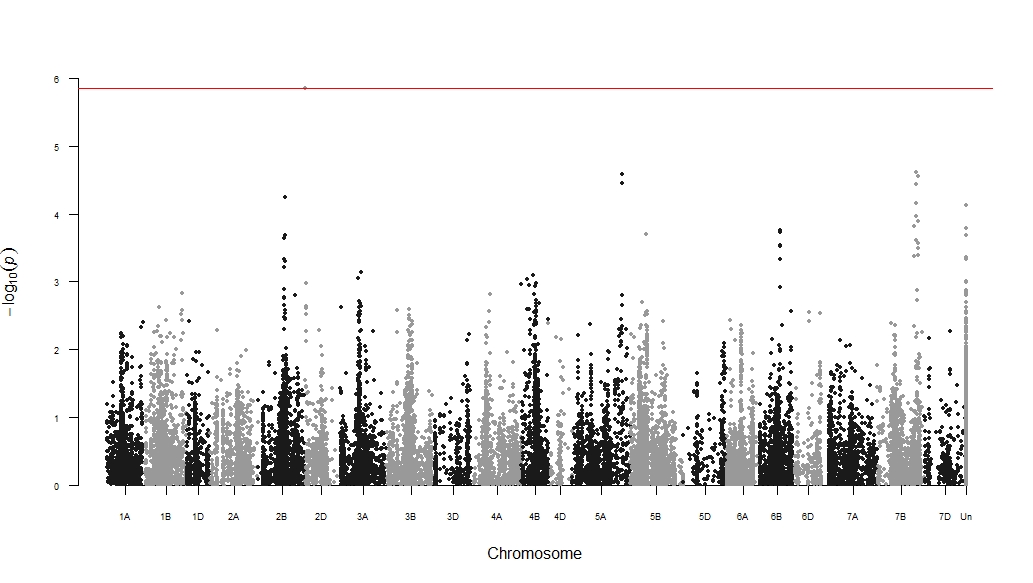

Supplement: S3 Fig — The red line represents the FDR corrected p-value of 0.04318, which is the closest to the threshold of FDR corrected p-value = 0.05 obtained in 2014. (JPEG) [file pone.0211730.s003.jpeg]

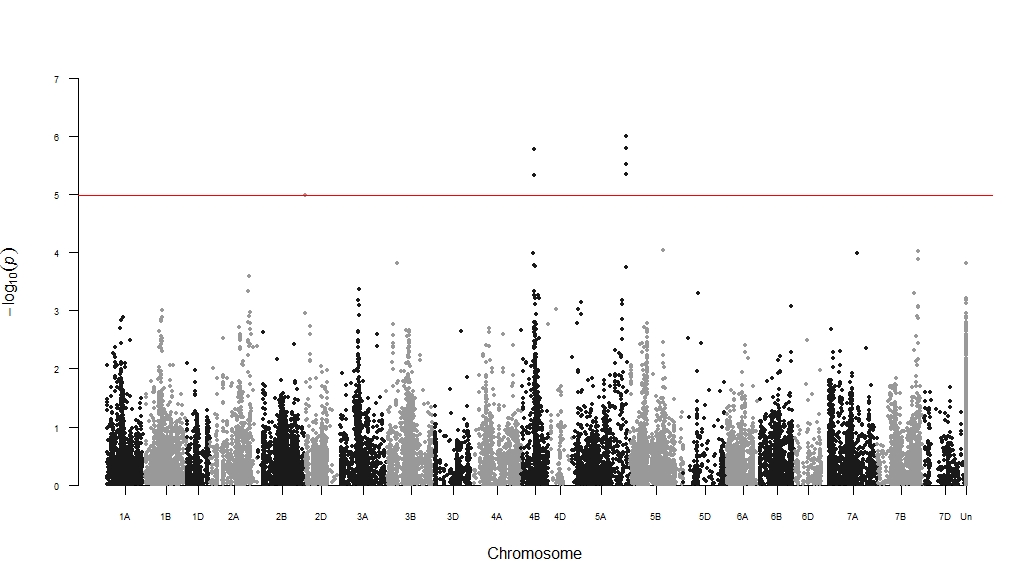

Supplement: S4 Fig — The red line represents the FDR corrected p-value of 0.04433, which is the closest to the threshold of FDR corrected p-value = 0.05 obtained in 2015. (JPEG) [file pone.0211730.s004.jpeg]

.Yield

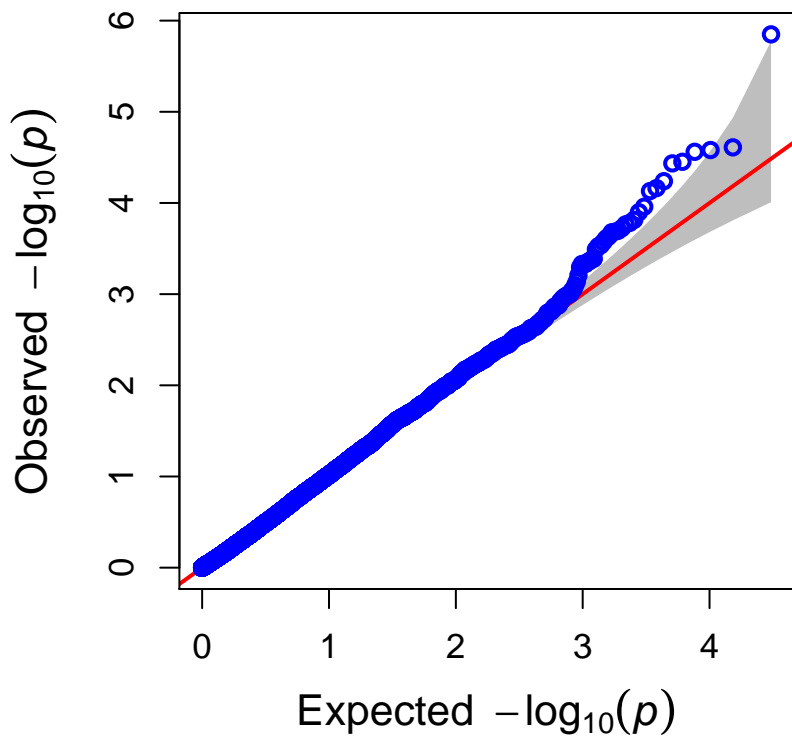

Supplement: S5 Fig — (PDF) [file pone.0211730.s005.pdf]

**.Yield**

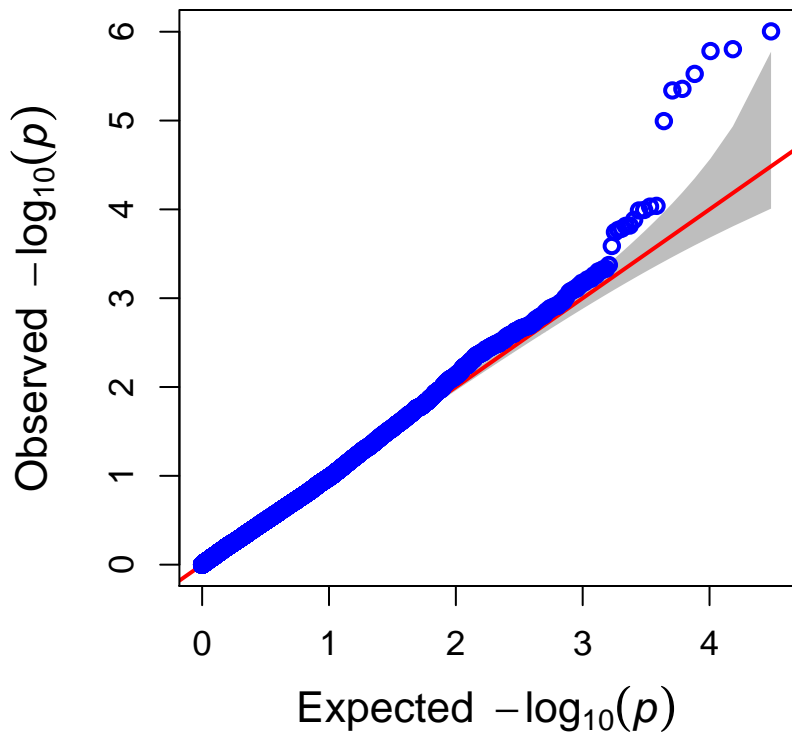

Supplement: S6 Fig — (PDF) [file pone.0211730.s006.pdf]
